# Cardiovascular risk reduction over time in patients with diabetes or pre-diabetes undergoing bariatric surgery: data from a single-center retrospective observational study

**DOI:** 10.1186/s12902-018-0317-4

**Published:** 2018-11-28

**Authors:** Matilde Rubio-Almanza, Rosa Cámara-Gómez, David Hervás-Marín, José Luis Ponce-Marco, Juan Francisco Merino-Torres

**Affiliations:** 1 Departamento de Endocrinología y Nutrición, Hospital Universitario y Politécnico La Fe, Valencia, Spain; 2 Unidad Mixta de Investigación en Endocrinología, Nutrición y Dietética clínica, Instituto de Investigación Sanitaria La Fe, Valencia, Spain; 3 Unidad de Bioestadística, Instituto de Investigación Sanitaria La Fe, Valencia, Spain; 4 Departamento de Cirugía Endocrina y Metabólica, Hospital Universitario y Politécnico La Fe, Valencia, Spain

**Keywords:** Bariatric surgery, RYGB, Type 2 diabetes, Pre-diabetes, CVD risk

## Abstract

**Background:**

Bariatric surgery is effective in remission of obesity comorbidities. This study was aimed at comparing CVD risk between morbidly obese patients with type 2 diabetes and pre-diabetes before and after bariatric surgery as well as assessing comorbidities.

**Methods:**

This is a retrospective observational study with 105 patients with type 2 diabetes (*DMbaseline*) and prediabetes (*preDMbaseline*) who underwent Roux-en-Y gastric bypass. Data were collected preoperative and then at 3,6,12,18,24,36,48, and 60 months after surgery. Anthropometric, cardiovascular and glycemic parameters were assessed. CVD risk was calculated using the Framingham Risk Score.

**Results:**

Prior to surgery, 48 patients had type 2 diabetes, while 57 had pre-diabetes. Mean age was 48 (9.2) and mean BMI was 52 (7.4). 26.1% of patients had a high CVD risk. CVD risk decreased in patients with type 2 diabetes and prediabetes at month 12 after surgery compared to the baseline risk (*p* < 0.001). BMI, body fat percentage, fasting plasma glucose, HbA1c, c-peptide, HOMA-IR, LDL-c, systolic blood pressure, and diastolic blood pressure decreased during the first year after surgery. From the 12th month until the 60th, they showed a flat trend, or a very mild increase in some cases. 3.2% of patients maintained high CVD risk at 60 months. Type 2 diabetes remission was 92%. No patient of the *preDMbaseline* group developed type 2 diabetes.

**Conclusion:**

Bariatric surgery reduces CVD risk in type 2 diabetes and pre-diabetes. Given that patients with type 2 diabetes benefit the most, more studies are necessary to consider pre-diabetes as a criterion for metabolic surgery in patients with BMI ≥ 35 kg/m^2^.

## Background

Obesity is a worldwide health problem, affecting children, adolescents as well as adults [1], being associated with comorbidities such as hypertension, dyslipidemia, type 2 diabetes, cancer, osteoarthritis and sleep apnea [2]. WHO (World Health Organization) reported diabetes global prevalence of 8.5% in 2014 [3]. It is well known that type 2 diabetes is associated with an increased risk of cardiovascular disease (CVD) [4] and recent evidence shows pre-diabetes behaves in a similar way [5]. Anthropometric measurements are useful for assessment of metabolic risk in overweight-obese subjects but not as markers of advanced atherosclerosis [6].

Type 2 diabetes is considered a criterion for bariatric surgery in patients with BMI > 35 kg/m^2^ but pre-diabetes is not included yet. Bariatric/metabolic surgery has shown to achieve high rates of type 2 diabetes remission in retrospective as well as prospective studies, thus reducing cardiovascular risk factors in patients with obesity [7]. Considering pre-diabetes a metabolic state between normoglycemia and type 2 diabetes, would be interesting to explore if bariatric surgery can reduce cardiovascular disease risk in this group of patients. The aim of this study was to compare cardiovascular risk between morbidly obese patients with type 2 diabetes and pre-diabetes before and after bariatric surgery and to evaluate the evolution of comorbidities.

## Methods

This retrospective observational study included morbidly obese patients. For analysis, we reviewed the electronic medical record of 105 patients with type 2 diabetes or pre-diabetes of a cohort of 313 obese patients, who had undergone Roux -en-Y Gastric Bypass (RYGB) surgery between June 2000 and November 2011 at Hospital Universitario y Politécnico La Fe, with a follow-up of 5 years. 70 of 105 patients completed a 5-year follow-up.

Inclusion criteria were BMI > 40 kg/m^2^, or above 35 kg/m^2^ with associated comorbidities (hypertension, type 2 diabetes, dyslipidemia, or OSA) who had previously tried lifestyle changes to control their obesity. All eligible subjects were aged 18–60 years and had passed a psychological evaluation (absence of drug and alcohol abuse and not having eating disorders). All surgical interventions had been performed by the same surgical team at our hospital and all patients signed an informed consent form before undergoing surgery.

Data were collected from electronic medical records at baseline (between 1 and 3 months prior to surgery) and at every follow-up visit (3, 6, 12, 18, 24, 36, 48, and 60 months after surgery). The following data were collected: blood pressure, anthropometric parameters (weight, height, waist circumference and body fat percentage), obstructive sleep apnea (OSA), and blood parameters (fasting plasma glucose, total cholesterol, LDL-c, HDL-c, triglycerides, glycated hemoglobin (HbA1c), plasma insulin and c-peptide). Normal or pathological values were based on cut-off parameters.

An oral glucose tolerance test in patients with unknown type 2 diabetes was done at baseline. Diabetes and cardiovascular medications were also considered.

All procedures in studies involving human participants were performed in accordance with the ethical standards of the institutional review board of Hospital Universitario y Politécnico La Fe and with the principles of the Declaration of Helsinki 2013. For this type of study formal consent was not required. This study was approved by the Ethics Committee of the Hospital Universitario y Politécnico La Fe.

### Study measures

The primary end point was to compare CVD risk between patients with type 2 diabetes and subjects with pre-diabetes before and 1 year after RYGB. To calculate the predicted 10-year risk of cardiovascular disease we used the Framingham Risk Score (FRS) [8], which requires age, sex, total and high-density lipoprotein cholesterol, smoking, systolic blood pressure, treatment for hypertension and diabetes status. The FRS was expressed as a percentage (%). A FRS > 20% was considered a high CVD risk.

Secondary end points were to describe the evolution of glycemic control, hypertension, dyslipidemia, OSA and weight parameters individually, for 5 years after surgery.

BMI was calculated as Kg/m^2^ and weight loss was expressed as total weight loss percentage (%TWL): follow-up weight - preoperative weight/ 100.

Excess weight loss percentage (%EWL) was calculated with the following formula:

(preoperative weight - follow-up weight/preoperative weight - ideal weight) × 100.

To calculate ideal body weight the equivalent to a BMI of 25 kg/m^2^ was used. An EWL ≥ 50% was considered successful weight loss [9].

Body fat percentage was measured by bio-electrical bio-impedance (Bodystar® 1500) once a year. Body fat percentage > 25% in men and > 33% in women was considered elevated [10].

Blood pressure was taken collecting the mean of three determinations in a seated state. Hypertension [11] was defined as: having a mean of at least 140/90 mmHg, or having a personal history of hypertension, or taking antihypertensive drugs. Dyslipidemia [12] was considered if LDLc was elevated (at least 160 mg/dl in pre-diabetes or 130 mg/dl in diabetes), or triglycerides > 150 mg/dl. Other criteria were having a previous personal history of dyslipidemia or taking lipid-lowering drugs. Criteria for diagnosing type 2 diabetes were one or more of the following conditions: fasting blood glucose level ≥ 126 mg/dl, HbA1c ≥ 6,5% glucose level after glucose tolerance test > 200 mg/l [13], or personal history of diabetes or taking hypoglycemic drugs. Pre-diabetes was considered in patients with fasting plasma glucose between 100 and 125 mg/dl, HbA1c between 5.7–6.4% or a glucose level between 140 and 199 mg/dl 2 h after a glucose tolerance test. Homeostatic Model Assessment of Insulin Resistance (HOMA-IR) ≥ 3.8 was considered as insulin resistance [14].

OSA was diagnosed through polysomnography before surgery. Improvement/resolution was considered after performing a new polysomnography and indicating the discontinuation of CPAP by the physician pneumologist after surgery. Remission of comorbidities was defined as the absence of disease according to the above criteria.

Information for relevant variables is shown at baseline and 6,12, 36 and 60 months. Follow-up data after surgery is presented according to diagnosis of type 2 diabetes (*DMbaseline*) or pre-diabetes (*preDMbaseline*) prior to surgery.

### Statistical analysis

Data were summarized using mean, standard deviation, median and first and third quartiles in the case of continuous variables and with relative and absolute frequencies in the case of categorical variables. Observations with missing data were excluded of the analysis. CVD risk was estimated using the formula in D'Agostino RB Sr et al. [8]. The association of glycemic status with CVD risk was assessed using a beta regression model including the interaction between fasting blood glucose levels and time in order to study the different trends over time between patients with high and low glucose levels. In order to ease the interpretation of the beta regression results, we provided marginal effect plots of the different fitted models depicting the estimated relationships between the different studied variables and CVD risk. Progression of other clinical variables such as BMI, HOMA-IR, LDL cholesterol, blood pressure, etc. was assessed using mixed regression models with splines to allow for non-linear trends in the data. All statistical analyses were performed using R (version 3.4.4) and R packages lme4 (version 1.1–15) and betareg (version 3.1–0).

## Results

### Baseline characteristics

The study included 105 patients (18 men and 87 women) who had undergone RYGB, 70 of whom (10 men and 60 women) had completed a 5 -year -follow-up. A summary of the most relevant baseline characteristics of our sample is presented in Table 1. The decrease in the number of patients in the follow-up was due to their referral to their local hospitals and their data could not be accessed. Mean age at surgery was 48 (9.2). Mean BMI was 52 (7.4) (the maximum and minimum were 35 and 77.7 kg/m^2^) and mean waist was 137.3 (15.2). Mean percentage body fat was 50.8% (7.3) and 100% of the patients had a high percentage of body fat.

**Table 1:** Baseline characteristics in obese morbid patients with type 2 diabetes (*DMbaseline*) and prediabetes (*preDMbaseline***)**

	*DMbaseline* (*n* = 48)	*preDMbaseline* (*n* = 57)
Weight (kg)	131.8 (122.5, 145.5)	126 (115, 141)
BMI (kg/m2)	53.5 (47.2, 57.1)	49.6 (45.7, 54.2)
Body fat (%)	53.1 (46.9, 55.4)	52.3 (47.2, 55)
Waist (cm)	137.5 (126.7, 150.2)	133.5 (125.8, 145)
Fasting plasma glucose (mg/dl)	145 (110.5, 175.5)	103 (96, 110)
Plasma insulin (μU/ml)	22 (15, 38)	26.4 (18, 33.8)
C-peptide (ng/ml)	4.8 (3.8, 6.2)	5 (3.8, 5.7)
HOMA-IR	8.5 (4.9, 14.3)	6.7 (4.7, 8.8)
HbA1c (%)	7 (6.1, 8.3)	5.5 (5.3, 5.9)
Total Cholesterol (mg/dl)	204 (176, 234.5)	204 (186, 229)
LDL-c (mg/dl)	128.5 (100.8, 155.5)	134 (113, 145)
HDL-c (mg/dl)	44 (36, 48)	45 (38, 53)
Triglycerides (mg/dl)	164 (125, 238)	133 (120, 172)
SBP (mm Hg)	140 (130, 155)	136 (120, 152)
DBP (mm Hg)	90 (80, 96)	83 (75.3, 96.5)
Hypertension (%)	44 (91.7%)	42 (73.7%)
Dyslipidemia (%)	39 (81.3%)	27 (47.4%)
10-year CVD risk (FRS) (%)	20 (12, 30)	7 (3, 14)

*Data are shown as median and 1st and 3rd quartiles. SBP Systolic blood pressure, DBP Diastolic blood pressure, FRS Framingham Risk Score, LDL-c LDL cholesterol, HDL-c: HDL cholesterol*

Other comorbidities, such as OSA, were present in 32.4% of the patients (19 patients of the *DMbaseline* group and 15 of the *preDMbaseline* group). 20% (21) of the patients were smokers.

Globally, according to the Framingham Risk Score, 26.1% (27) of the patients had a high CVD risk (a 10-year risk of a CVD event > 20%).

Prior to surgery, 48 patients (45.7%) had type 2 diabetes. A total of 23 patients were diagnosed of type 2 diabetes at baseline (16 of whom after a glucose tolerance test). 50% of the patients with diabetes took medications (18 patients took 1 drug and 6 took 2 drugs). Metformin was the most common prescribed, and only 3 patients required insulin.

Fifty-seven (54.2%) patients of the cohort had pre-diabetes. A HOMA-IR index > 3.8 was detected in 77.1% (81) of all of patients. C-peptide levels were similar in both groups.

Other comorbidities, such as hypertension, were present in 81.9% (86) of the patients at baseline. 57% of the patients took 1 antihypertensive drug, and about 20% of the patients took 2 or 3 medications. Only 1 patient was treated with 4 drugs.

62.9% (66) of the patients had dyslipidemia, and 19.7% (13) of whom took treatment prior to intervention.

### Association between CVD risk and fasting glucose levels

**Fig. 1:**
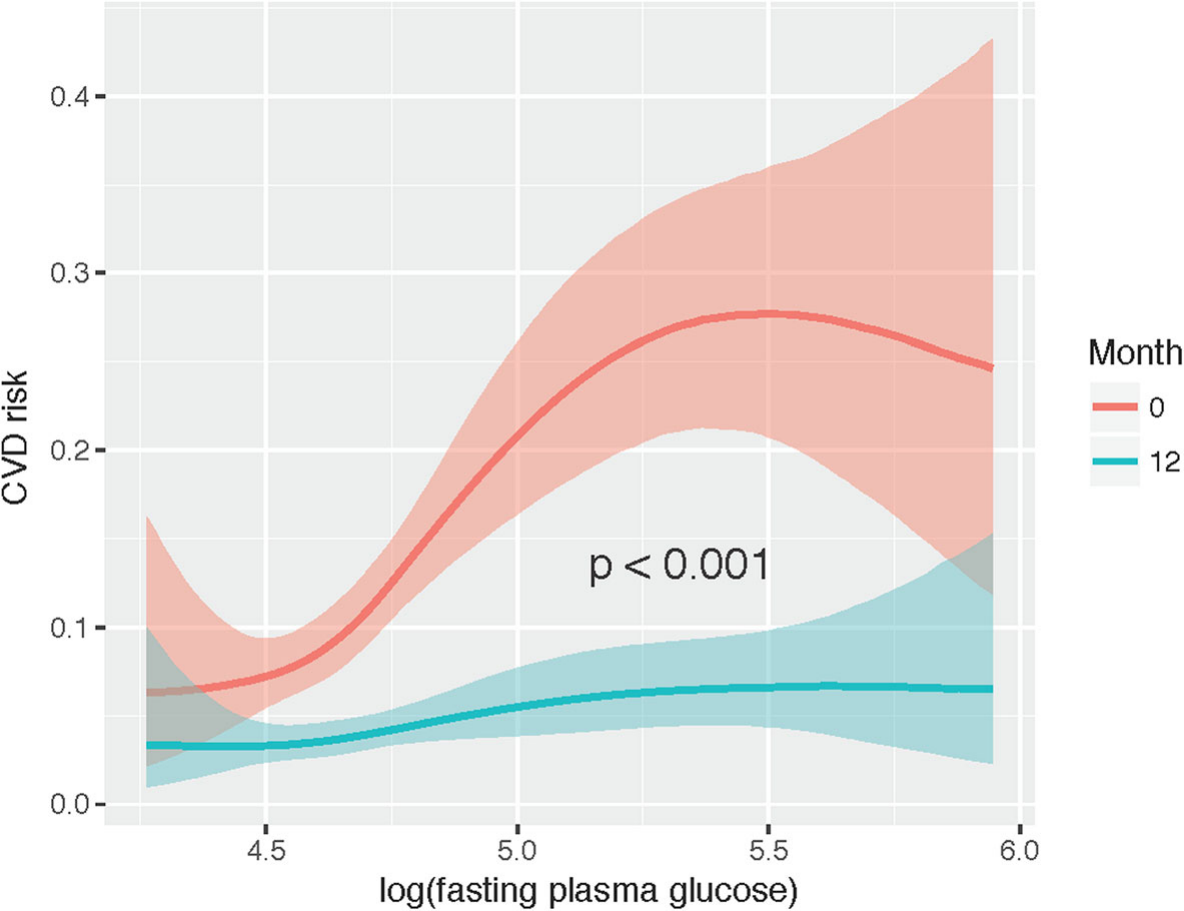
Marginal effects plot of the model explaining the relationship between fasting glucose levels and CVD risk at baseline and at 12 months. On the X-axis, the logarithm of the fasting plasma glucose levels is represented for baseline (*red*) and for month 12 (*blue*). The Y-axis represents the CVD risk in scale 0–1. *P*-value refers to the interaction between fasting plasma glucose levels and month

The primary end point was assessed in 105 patients. Progression of CVD risk over time and its association with fasting glucose levels were studied using a beta regression model. The model showed a clear effect of fasting glucose levels on the different progression of CVD risk over time (Fig. 1). Higher glucose levels at baseline were clearly associated with a higher CVD risk (OR = 4.35 [2.73, 6.99], *p* < 0.001) and the effect. The difference between baseline and month 12 was also statistically significant (OR = 0.31 [0.26, 0.36], p < 0.001). Nevertheless, after the intervention all patients reached similar levels of CVD risk regardless of their previous status. Therefore, patients with higher fasting glucose levels are the ones that benefit the most from the intervention regarding CVD risk (OR = 0.44 [0.27, 0.71], *p* < 0.001).

**Fig. 2:**
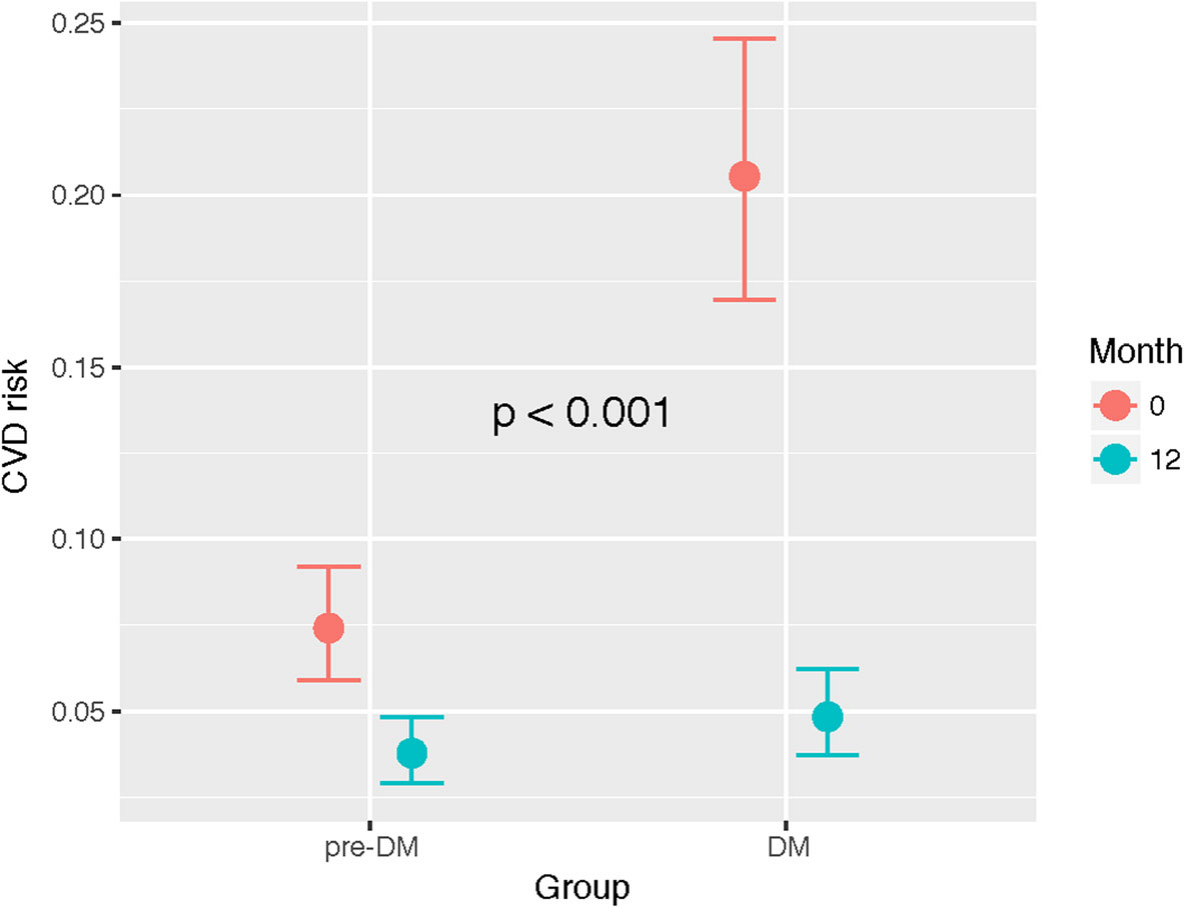
Marginal effects plot of the model explaining the relationship between diabetes/pre-diabetes status and CVD risk at baseline and at 12 months. On the X-axis, the two groups of patients are represented for baseline (*red*) and for month 12 (*blue*). The Y-axis represents the CVD risk in scale 0–1. *P*-value refers to the interaction between diabetes status and month

The main analysis was performed using fasting glucose levels as a continuous numerical variable, but the same analysis was also performed after classifying patients with type 2 diabetes or pre-diabetes. Results of this analysis were similar to those obtained using the continuous fasting glucose levels: patients with type 2 diabetes experienced a clear benefit after the intervention compared to pre-diabetes (Fig. 2). At baseline, patients with type 2 diabetes showed a statistically significant higher CVD risk compared to patients with pre-diabetes (OR 3.23 [2.32, 4.50], *p* < 0.001).

Patients with pre-diabetes showed a significant reduction in risk (OR: 0.49 [0.40, 0.60], p < 0.001) 12 months after surgery, compared to their baseline risk. Nevertheless, at that time point (month 12), patients with type 2 diabetes showed a larger reduction in CVD risk than those with pre-diabetes (OR 0.40 [0.30, 0.63], p < 0.001).

### Evolution of the different secondary clinical parameters after surgery

**Fig. 3:**
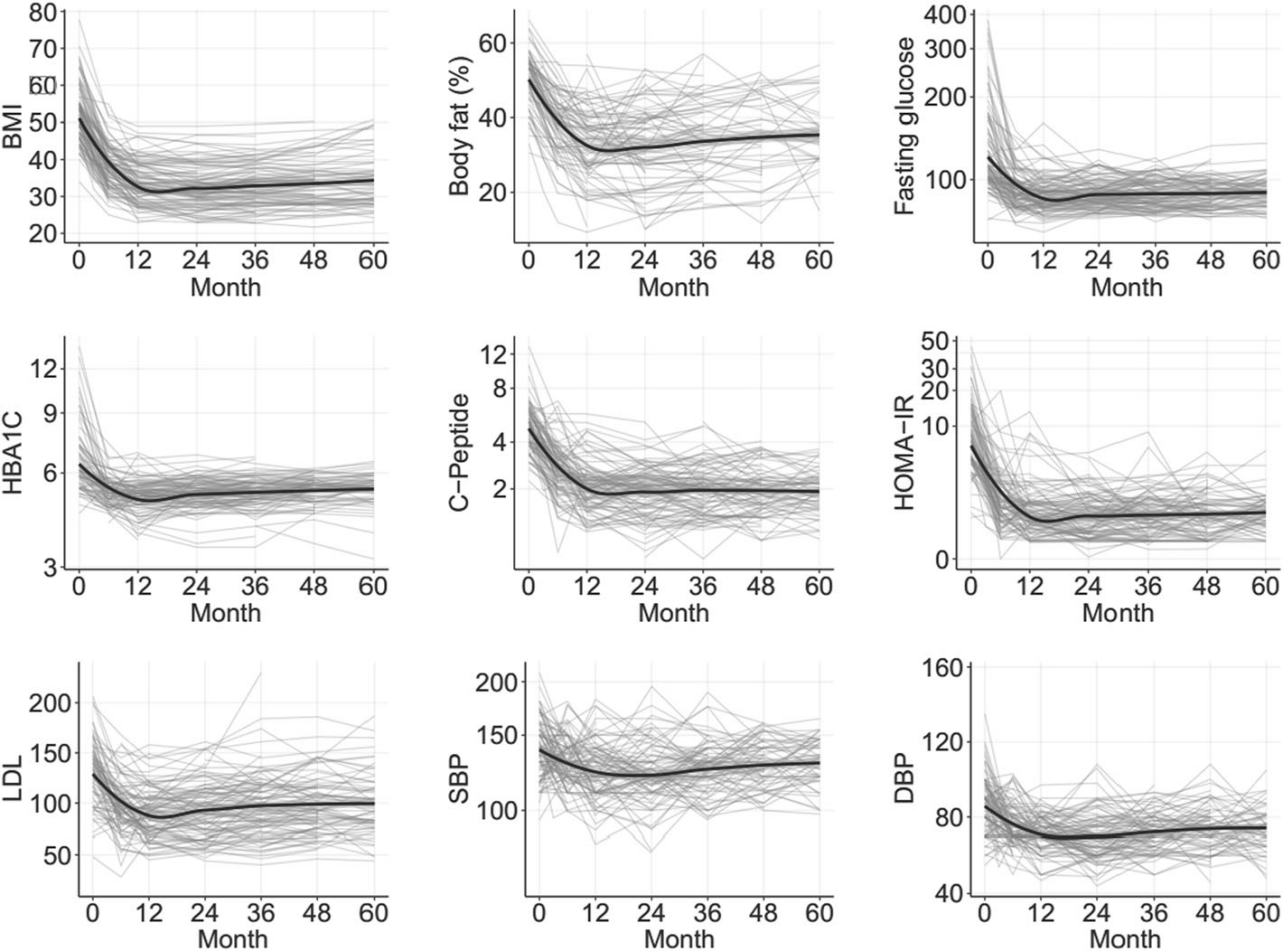
Trends over the first 60 months after the intervention for the different parameters studied (BMI, % body fat, fasting glucose, HBA1C, C-peptide, HOMA-IR, LDL cholesterol, systolic blood pressure (SBP), and diastolic blood pressure (DBP)). Average trend is represented by a black line, individual trends for each patient are represented by gray lines. *P* < 0.001

In order to gain more insight into the different outcomes related to the intervention in this type of patients, the trends over time for eight different parameters were studied (BMI, % body fat, fasting glucose, HbA1c, C-peptide, HOMA-IR, LDL-c, systolic and diastolic blood pressure). These trends are depicted in Fig. 3. In general, all parameters display a similar behavior: from the 12th month until the 60th, they showed a flat trend, or a very mild increase in some cases. There were no statistically significant differences in evolution of BMI, body fat percentage, SBP and DBP between the *DMbaseline* group and the *preDMbaseline* group. Median and quartiles of evolution of parameters described at baseline are shown in Table 2.

**Table 2:** Evolution of clinical parameters after bariatric surgery (6, 12, 36, 60 months) in *DMbaseline* group and *preDMbaseline* group

	*6 months*		*12 months*		*36 months*		*60 months*	
	*DMbaseline (n = 46)*	*preDMbaseline* *(n = 52)*	*DMbaseline* *(n = 48)*	*preDMbaseline* *(n = 57)*	*DMbaseline* *(n = 46)*	*preDMbaseline* *(n = 55)*	*DMbaseline* *(n = 32)*	*preDMbaseline* *(n = 38)*
Weight (kg)	98.5 (85, 105.7)	95.8 (83.9, 103.3)	84.3 (77, 100.5)	83 (72.3, 94)	82.8 (75.8, 99.3)	84.8 (70.6, 93.8)	85.3 (77, 105.8)	77.1 (70.5, 91.9)
BMI (kg/m^2^)	38.3 (33.9, 40.7)	37 (33.9, 41.3)	33.7 (30.8, 38.4)	32.6 (28.6, 37.7)	32.5 (29.3, 37)	33 (28.3, 37.4)	34.5 (29.8, 39.4)	32 (28.8, 36.7)
Body fat (%)	38.4 (32, 42.6)	39.6 (33.4, 43.1)	34.5 (27.2, 38.3)	34.5 (26.9, 39.3)	36.1 (25.4, 41.6)	32.7 (24.6, 42)	35.6 (32.4, 37.1)	33.6 (28.5, 45.5)
Waist (cm)	116 (103, 126.3)	111 (104, 122.5)	108 (98, 117)	104 (97.3, 112.5)	105.5 (98.5, 116.3)	104 (95, 113)	103 (97.5, 120.3)	102 (91, 111)
Fasting plasma glucose (mg/dl)	96 (85.8, 109.3)	87 (79, 93)	91 (80.8, 99.3)	83 (79, 90)	92 (82, 100.8)	84.5 (80.3, 92.8)	91.5 (86.8, 99.8)	86 (80, 94)
Plasma insulin (μU/ml)	7 (4, 12)	8 (5, 11.7)	6 (4, 8.2)	5 (3.9, 7.2)	5 (3, 7.4)	5.7 (4, 8)	6 (4, 8)	6 (4, 8)
C-peptide (ng/ml)	2.8 (2, 3.5)	2.6 (2.2, 3.1)	2 (1.6, 2.6)	2 (1.7, 2.4)	2 (1.4, 2.5)	2 (1.5, 2.4)	1.9 (1.5, 2.6)	2 (1.5, 2.3)
HOMA-IR	1.8 (1.1, 3.3)	1.6 (1.1, 2.4)	1.4 (0.9, 1.9)	1.1 (0.7, 1.6)	1.1 (0.7, 1.7)	1.1 (0.8, 1.8)	1.4 (1, 1.9)	1.2 (0.8, 1.8)
HbA1c (%)	5.3 (5.1, 5.6)	5 (4.8, 5.4)	5.3 (5, 5.6)	5.2 (4.8, 5.3)	5.4 (5.2, 5.7)	5.1 (4.9, 5.4)	5.7 (5.4, 5.9)	5.3 (4.9, 5.6)
Total Cholesterol (mg/dl)	166 (145, 180)	163 (143.5, 180.5)	160.5 (144.8, 178.5)	155 (137.3, 175.3)	178 (158.3, 190)	167.5 (142, 184.8)	177 (162.8, 196)	175 (146, 194)
LDL-c (mg/dl)	97 (81, 109)	94 (84, 111)	94 (75, 107.5)	85 (71.8, 100.3)	104.5 (84.3, 119)	88.5 (71.3, 109.3)	104 (89, 114.5)	89 (76, 112)
HDL-c (mg/dl)	41 (36, 46)	43 (38.5, 50.5)	48 (42, 52.5)	50.5 (43, 60)	53 (44, 60)	56 (48, 69.8)	56 (49, 64)	61 (51, 72)
Triglycerides (mg/dl)	117 (91, 157)	102 (82, 127)	93 (75, 124.5)	82 (67, 108.3)	93 (72.8, 119.3)	86 (72.3, 118.8)	97 (76, 116)	89 (66, 108)
SBP (mm Hg)	130 (120, 150)	126 (119, 138)	123 (110, 136)	120 (110, 137)	130 (122, 140)	120 (117, 130)	131 (120, 140)	130 (119, 144.5)
DBP (mm Hg)	72 (70, 81.8)	71 (70, 80)	72 (69.5, 77.5)	70 (65, 79)	75 (68.8, 78.5)	70 (63, 78)	76 (70, 81.3)	70 (64.5, 79)

Data are shown as median and quartiles. *SBP* systolic blood pressure, *DBP* Diastolic blood pressure, *LDL-c* LDL cholesterol, *HDL-c* HDL cholesterol

### Final evaluation 5 years after surgery

Seventy patients completed the 5-year-follow up. Among them, dyslipidemia was present in 9.4% (3) of patients of the *DMbaseline* group, and 15.8% (6) of patients of the *preDMbaseline* group. Only 2 subjects of our cohort took lipid-lowering treatment at 60 months.

Hypertension resolved in 50% (16) of patients of the *DMbaseline* group, and 57.9% (22) of the *preDMbaseline* group. 18 patients of our cohort took antihypertensive treatment (13 of the *DMbaseline* group and 5 of the *preDMbaseline* group). Most of them were treated with 1 drug. Hypertension worsened in one patient (treatment with 5 drugs).

According to the Framingham Risk Score, 3.2% (2) of the patients maintained a high CVD risk (a 10-year risk of a CVD event > 20%) five years after surgery. Median of %FRS was 5 (3, 10) in the *DMbaseline* group, and 5 (2, 7) in the *preDMbaseline* group.

OSA was present in 7.9% (3) of the *preDMbaseline* group, and 6.3% (2) of the *DMbaseline* group.

Regarding weight parameters, mean %TWL and %EWL were 32.8 and 63.4 in the *DMbaseline* group and 34 and 69.5 in the *preDMbaseline* group, respectively, one year after surgery. We did not find statistically significant differences in mean %TWL and %EWL between both groups 1 and 5 years after surgery. Successful weight loss was achieved in 71.9% (23) of the *DMbaseline* group and 83.3% (32) of the *preDMbaseline* group.

At 60 months, only 4 patients had type 2 diabetes and 20 patients had pre-diabetes (they all belonged to the *DMbaseline* group). The type 2 diabetes remission rate was 92% (29/32). The mean decrease in HbA1c was 2.05 in the *DMbaseline* group and 0.37 in the *preDMbaseline* group. No patient of the *preDMbaseline* group developed type 2 diabetes. Medications were reduced after intervention and only 2 subjects continued to take hypoglycemic drugs.

The mortality rate after surgery was 0% in our cohort of patients. Gastrointestinal bleeding was present in 3% of cases and there were 3 cases of Dumping syndrome.

## Discussion

Our study shows that Roux-en-Y gastric bypass surgery decreases the cardiovascular disease risk, leading to a sustained weight loss and improvement of glycemic control, hypertension, and dyslipidemia in patients with type 2 diabetes and pre-diabetes over 60 months after the intervention.

Cardiovascular disease risk was elevated in our cohort at baseline (26.1% of all patients had a 10-year risk of a CVD event > 20%). As previously shown, the higher the fasting plasma glucose, the higher the CVD risk. After intervention, the CVD risk decreased in both groups (type 2 diabetes and pre-diabetes) 12 months after surgery with a clear benefit in type 2 diabetes compared to pre-diabetes (Fig. 2). Many observational studies have demonstrated that bariatric surgery reduces cardiovascular disease in obese patients (myocardial infarction, stroke, cardiovascular events, and mortality) compared to non-surgical controls [15–17]. Recent evidence showed a lower CVD risk in patients with type 2 diabetes who underwent Roux-en-Y gastric bypass surgery [18]. Other non-surgical treatment available such as Lorcaserine, facilitated weight loss in overweight or obese patients without a high rate of major cardiovascular events. Additionally, this drug has shown to decrease the risk of incidence of diabetes and microvascular complications and to induce remission of hyperglycaemia [19, 20].

A relevant change in the CVD risk can be observed in the *DMbaseline* group, but the improvement in the CVD risk in the *preDMbaseline* group should also be highlighted. It is known that hyperglycemia, beyond oxidative stress, can affect vasculature cells at an early stage, necessitating early treatment, even in prediabetes [21]. *Echouffo-Tcheuqui JB* et al. [22] showed individuals with early-onset pre-diabetes, despite lifelong avoidance of overt diabetes, had more propensity for death due to cardiovascular disease compared with those who maintained lifelong normal glucose levels. Despite this evidence, patients with type 2 diabetes benefit the most from surgery and much more studies are needed to consider pre-diabetes itself a comorbidity of sufficient weight to be included as a criterion for surgery in patients with BMI > 35. CVD risk estimated with FRS is strongly influenced by the presence of diabetes. Thus much of the higher benefit of bariatric surgery found from the studies among subjects with diabetes versus those without, is derived from the remission of diabetes status. While changing in other metabolic trait were similar in the two groups.

The assessment of individual cardiovascular risk factors (hypertension and dyslipidemia) after bariatric surgery also confirms the beneficial effects of metabolic surgery. An improvement was observed in both groups (*DMbaseline and preDMbaseline* group) with a sharp decrease during the first year and a flat trend until 60 months after surgery. The potential mechanisms involve a reduction in systemic inflammation and the restoration of a normal adipokine secretory profile [16]. A recent study suggests that some of the effects of bariatric surgery take place speedily after surgery independent of weight loss and may involve increased HDL levels [23, 24], which was the case in both groups after intervention. This increase is thought associated with improvement in hepatic insulin sensitivity [25].

No statistically significant differences were found in the evolution of SBP, DBP, LDLc, BMI, and body fat percentage between the *DMbaseline* and the *preDMbaseline* groups, probably because the two groups became similar after surgery. Baseline plasma insulin and c-peptide levels and its evolution in both groups were similar after surgery.

Several observational studies and randomized controlled trials reported a type 2 diabetes remission rate between 36 and 78% [7, 26, 27] after surgery. Our results showed a high remission rate in patients with type 2 diabetes (92%) 5 years after surgery with RYGB, and no patient with pre-diabetes developed type 2 diabetes. Similar outcomes in resolution of type 2 diabetes and obesity comorbidities were reported with other surgical procedures such as VSG [28].

This study has a few limitations: as other longitudinal studies, this is a retrospective observational study. In our cohort, median BMI was elevated in both groups, especially in the *DMbaseline* group. Many patients were initially included with bariatric criteria (BMI ≥ 40) and were diagnosed with type 2 diabetes or pre-diabetes in the pre-surgery evaluation. The *DMbaseline* group had an acceptable glycemic control, likely to lead to a high diabetes remission after surgery. Moreover, 35 patients had been referred to their local hospitals, which resulted in the loss of their data.

## Conclusions

Our results support the beneficial effects of metabolic surgery decreasing cardiovascular risk in type 2 diabetes as well as pre-diabetes, and improving glycemic control and obesity comorbidities in both groups. A greater benefit among subjects with diabetes as compared to those with pre-diabetes was observed in our study. More studies are necessary to consider pre-diabetes as a criterion for metabolic surgery in patients with BMI ≥ 35 kg/m^2^.

## Abbreviations

%EWL: Excess weight loss percentage
%TWL: Total weight loss percentage
BMI: Body mass index
CPAP: Continuous Positive Airway Pressure
CVD: Cardiovascular disease
DBP: Diastolic blood pressure
FRS: Framingham Risk Score
HbA1c: Glycated hemoglobin
HDL-c: HDL cholesterol
LDL-c: LDL cholesterol
OSA: Obstructive sleep apnea
RYGB: Roux -en-Y Gastric Bypass
SBP: Systolic blood pressure
